# Therapeutics of the Eye

**Published:** 1897-02

**Authors:** 


THERAPEUTICS OF THE EYE. By Chas. C. Boyle, M. D., O. et A. Chir. Surgeon to the New York Ophthalmic Hospital; Professor of Ophthalmic and Aural Therapeutics in the College of the N. Y. Ophthalmic Hospital, etc., etc. Published by Boericke, Runyon & Ernesty, 497 Fifth Avenue, N. Y. Mailed upon receipt of price: Cloth binding $3.50 net; half morocco $4.50 net.

This book is cordially recommended to the homœopathic profession for its treatment of eye diseases from the homœopathic standpoint.

It is very different from the usual productions of this kind claiming to teach the principles of the new school.

It is strictly homœopathic, and gives the leading indications of the best remedies for eye troubles with great clearness.

It is divided into three parts. The first part gives the pathogenesis of the remedies each under its own name.

The second part, called "Applied Therapeutics," gives the leading indications of the most important remedies under the name of the anatomical part and function. Thus we have chapters on the conjunctiva, the cornea, the iris and ciliary body, the retina and optic nerve, the vitreous body, the lens, the sclera, the lids, and the lachrymal apparatus. Then we have indications for disturbances of vision, for glaucoma, for disturbances of the action of the muscles, and for pain. Asthenopia is treated of in the giving of indications for remedies very copiously.

The third part contains a repertorial index which is at once a repertory to the remedies and an index to the book. This index is very good, yet it is lacking in one thing, and that is a system of cross references. Thus under the letter "D" we have "dim vision," and the indications here are very full. Then under "Vision" we have a sub-division "dim" which is very scant. Here there should be a cross reference to "Dim Vision," that the reader might instantly get all there is in the book on this point. Such cross references which we have noticed the need of in one or two places, would add greatly to the value of the book. The printing is very handsome and in approved homœopathic style.

This style is now regarded as indispensable to homœopathic text-books that are expected to be of daily use to the active practitioner.

The volume contains 404 pages, of which the pathogenesis of the remedies occupies 150 pages. The "Applied Therapeutics" includes 164 pages, and the repertory the remaining 80 pages. Surgical treatment is not considered, and need not be, as that can be found in so many other books which neglect purely medicinal treatment.

